# An Exploratory Study of Serum 25-Hydroxyvitamin D Concentration and Psychological Distress Among Aboriginal and Torres Strait Islander Peoples in Australia

**DOI:** 10.3390/nu18101563

**Published:** 2026-05-14

**Authors:** Belinda Neo, Noel Nannup, Dale Tilbrook, Carol Michie, Cindy Prior, Eleanor Dunlop, Brad Farrant, Won Sun Chen, Carrington Shepherd, Lucinda Black

**Affiliations:** 1 Curtin Medical School, Curtin University, Kent Street, Bentley, WA 6102, Australia; 2 The Kids Research Institute Australia, Nedlands, WA 6009, Australia; 3 Maalinup Aboriginal Gallery, Caversham, WA 6055, Australia; 4 Curtin School of Population Health, Curtin University, Kent Street, Bentley, WA 6102, Australia; 5 Institute for Physical Activity and Nutrition (IPAN), School of Exercise and Nutrition Sciences, Deakin University, Geelong, VIC 3220, Australia; 6 Ngangk Yira Institute, Murdoch University, Murdoch, WA 6150, Australia

**Keywords:** Aboriginal and Torres Strait Islander peoples, vitamin D, serum 25(OH)D concentration, psychological distress, mental health

## Abstract

Background/Objectives: The colonisation of Australia around 250 years ago left a significant enduring impact on the mental health of Aboriginal and Torres Strait Islander peoples. Vitamin D may play a role in modulating mental health as its receptors are present in the brain regions associated with mood and behaviour regulation. We aimed to conduct an exploratory study to investigate associations between serum 25-hydroxyvitamin D (25(OH)D) concentration and Kessler Psychological Distress Scale 5 (K5) [low/moderate *vs*. high/very high psychological distress] among Aboriginal and Torres Strait Islander peoples. Methods: We used cross-sectional data from the 2012–2013 Australian Aboriginal and Torres Strait Islander Health Survey. Binary logistic regression was used to test associations between serum 25(OH)D concentration and K5, adjusting for age, sex, education, remoteness, socioeconomic status, season, alcohol intake, and smoking (*n* = 1983). Results: There was no association between serum 25(OH)D concentration and K5 in the total population. In our exploratory analyses, higher serum 25(OH)D concentration (per 10 nmol/L) was significantly associated with 10% lower odds of high/very high levels of psychological distress among females. When stratified by remoteness, higher serum 25(OH)D concentration (per 10 nmol/L) was significantly associated with 11% lower odds of high/very high levels of psychological distress among those living remotely. Conclusions: The findings of this study suggest no association between serum 25(OH)D and K5 among the total population, but with some exploratory evidence of differences by sex and remoteness. Given the high prevalence of low vitamin D status among this population, promoting adequate vitamin D status remains an important public health issue.

## 1. Introduction

Aboriginal and Torres Strait Islander peoples have inhabited Australia for approximately 65,000 years [1]. The colonisation of Australia ~250 years ago resulted in the dispossession of land and forced removal of children and babies from Aboriginal and Torres Strait Islander families. The aftermath of colonisation has left a significant enduring impact on the mental health and wellbeing of Aboriginal and Torres Strait Islander peoples [2,3]. Findings from the 2018–2019 National Aboriginal and Torres Strait Islander Health Survey (NATSIHS) showed that 17% of Aboriginal and Torres Strait Islander peoples aged ≥ 2 years experienced anxiety, and 13% experienced depression/feelings of depression [4]. A higher proportion of females (35%) experienced high/very high levels of psychological distress compared to males (26%) [4].

Low vitamin D status is prevalent worldwide [5], including among Aboriginal and Torres Strait Islander peoples; serum 25-hydroxyvitamin D (25(OH)D) concentration was < 50 nmol/L among 27% of Aboriginal and Torres Strait Islander adults aged ≥ 18 years in 2012–2013 [6]. The prevalence of serum 25(OH)D concentration < 50 nmol/L was higher among those living in remote areas (39%) compared to those in non-remote areas (23%) [6]. Vitamin D may have a role in modulating mental health conditions as its receptors are present in brain regions involved in the pathophysiology of stress and mood disorders, such as depression and anxiety [7,8,9]. The postulated mechanisms of vitamin D on mental health conditions include the modulation of neurotrophic factors necessary for neuron viability and growth, synthesis of neurotransmitters responsible for mood regulation, and modulation of the immune system [9,10,11]. These mechanisms suggest that vitamin D status may be a modifiable risk factor for mental health. 

Among various population groups, findings from observational studies testing associations between serum 25(OH)D concentration and mental health conditions, such as depression, have shown inconsistent findings: some studies showed an inverse association [12,13,14], while others showed no association [15,16,17]. An umbrella meta-analysis of cohort studies (*n* = 5) reported that lower serum 25(OH)D concentration was significantly associated with higher odds of depression [18]. In the same review, an umbrella meta-analysis of randomised controlled trials (*n* = 10) reported a decrease in depression symptoms among participants receiving vitamin D supplementation compared with placebo [18]. Notably, most previous studies examining the relationship between serum 25(OH)D concentration and mental health have focused on depression, with limited evidence for other mental health outcomes. To our knowledge, no published studies have examined associations between serum 25(OH)D concentration and mental health among Aboriginal and Torres Strait Islander peoples in Australia. 

In the 2012–2013 Australian Aboriginal and Torres Strait Islander Health Survey (AATSIHS), mental health was assessed using the Kessler Psychological Distress Scale 5 (K5), and serum 25(OH)D concentration was measured using an internationally certified liquid chromatography with tandem mass spectrometry (LC-MS/MS) assay [19,20]. Given the potential role of serum 25(OH)D concentration in mental health, we aimed to conduct an exploratory study to investigate associations between serum 25(OH)D concentration and psychological distress among Aboriginal and Torres Strait Islander peoples in Australia.

## 2. Materials and Methods

### 2.1. Project Governance

Aboriginal and Torres Strait Islander Elders and researchers are valuable knowledge holders who provide critical cultural context and guidance for research. We collaborated with Aboriginal and Torres Strait Islander Elders and researchers throughout this project to ensure that our reporting was respectful and reflective of their perspectives. Ethics approval for this study was granted by the Western Australian Aboriginal Health Ethics Committee (HREC979). The interview components of the AATSIHS were conducted under the Census and Statistics Act 1905. The biomedical component of the AATSIHS was collected under the Privacy Act 1988. At the national level, ethics approval for the biomedical component of the AATSIHS was granted by the Australian Government Department of Health and Ageing’s Departmental Ethics Committee. Ethics approval for the biomedical component of the AATSIHS was granted at the jurisdictional level for New South Wales, South Australia, Western Australia, Northern Territory, and Queensland Health Service Districts [19]. Those participating in the biomedical component of the AATSIHS provided informed written consent.

### 2.2. Study Population

We used cross-sectional data from the 2012–2013 AATSIHS conducted from April 2012 to July 2013, which included data from the 2012–2013 NATSIHS and biomedical data from the 2012–2013 National Aboriginal and Torres Strait Islander Health Measures Survey [19,21]. The 2012–2013 AATSIHS was conducted across Australia and included Aboriginal and Torres Strait Islander peoples aged ≥ 2 years living in non-remote and remote areas. Of the 6701 households approached for the 2012–2013 NATSIHS, 5371 (80.2%) households, with a total of 9317 participants, provided adequate responses. Comprehensive information was collected, including demographics, socioeconomic status, and mental health [19].

Participants were interviewed by trained interviewers from the Australian Bureau of Statistics (ABS), and data were recorded electronically using a Computer Assisted Interview instrument [19]. In non-remote areas, up to two adults (aged ≥ 18 years) and up to two children per household were selected to participate in the interview. In remote areas, one adult and/or one child per household were selected to participate in the interview. Children aged 15–17 years were interviewed in person if a parent or guardian granted permission; if permission was not granted, an adult answered questions on their behalf. 

Participants aged ≥18 years who participated in the 2012–2013 NATSIHS were invited to provide a blood sample for the measurement of biomarkers. Blood samples were collected at Sonic Healthcare clinics, from home visits, or temporary clinics at the Aboriginal Medical Services [22]. For regional areas in South Australia and the Northern Territory, other pathology services, such as the Institute of Medical and Veterinary Science Pathology, were also used to collect blood samples [22].

### 2.3. Covariates

The ABS reported age as a continuous variable for people aged 0–64 years. Due to the small number of participants in older age groups, the ABS reported only categorical data for those aged > 64 years, as follows: 65–69 years, 70–74 years, and ≥ 75 years. We assigned 67 years for all participants aged 65–69 years, 72 years for all participants aged 70–74 years, and 75 years for all participants aged ≥ 75 years [23].

The ABS classified smokers into five categories: current smokers who smoke daily, current smokers who smoke weekly, current smokers who smoke less than weekly, ex-smokers, and never smoked (an individual who had never smoked or had smoked < 100 cigarettes or < 20 pipes, cigars, or other tobacco products). We re-categorised participants into two groups: (i) ex/non-smokers, and (ii) current smokers. The average daily alcohol intake was calculated by the ABS based on the reported type, number, and size of alcoholic drinks consumed on each day over the last three drinking days. The ABS reported alcohol intake by standard drinks (10 g or 12.5 mL of alcohol) as a continuous variable.

We regrouped the education categories provided by the ABS into three categories: (i) no/primary/high school, (ii) diploma/certificate, and (iii) university. The ABS reported socioeconomic status in deciles according to the 2011 Index of Relative Socioeconomic Disadvantage [19]. We regrouped deciles into quintiles. A lower quintile indicated a greater overall disadvantage; a higher quintile indicated a lesser overall disadvantage. The ABS classified the location of residence as non-remote or remote, according to the Australian Statistical Geography Standard [19].

It was not possible to translate the month of blood collection into the traditional calendars of the Aboriginal and Torres Strait Islander peoples. Hence, as a proxy measure of the ultraviolet-B radiation across the year, we categorised the month of blood collection using the Western calendar definitions of Australian seasons: spring (September–November), summer (December–February), autumn (March–May), and winter (June–August) [24].

### 2.4. Psychological Distress

Participants aged ≥ 18 years were interviewed using the K5 to assess their levels of negative emotional states over the preceding four weeks [19]. The K5 is a 5-item questionnaire, condensed and modified from the Kessler Psychological Distress Scale 10. The K5 was developed in consultation with Aboriginal and Torres Strait Islander peoples and endorsed by the original developer of the scale [19,25,26]. Each item in the questionnaire is based on a five-level response scale (5: all of the time, 4: most of the time, 3: some of the time, 2: a little of the time, 1: none of the time). The total score is calculated as the sum of the scores from all five questions, with possible scores ranging from 5 to 25. A score of 5 to 11 indicates low/moderate levels of psychological distress, and a score of 12 to 25 indicates high/very high levels of psychological distress. 

### 2.5. Measurement of Serum 25(OH)D Concentration

Blood samples were sent to the Douglass Hanly Moir pathology laboratory (Sydney, New South Wales) for measurement of serum 25(OH)D concentration using an LC-MS/MS assay that was certified to the reference measurement procedures developed by the National Institute of Standards and Technology, Ghent University, and Centers for Disease Control [19,20]. The ABS reported serum 25(OH)D concentration ≤ 15 and ≥ 130 nmol/L as 15 and 130 nmol/L, respectively.

### 2.6. Statistical Analysis

All statistical analyses were conducted in the secure research environment provided by the ABS [27]. Statistical analyses were performed using Stata Statistical Software (Version 18) [28]. We incorporated the survey weight supplied by the ABS, which weighted the data to the Aboriginal and Torres Strait Islander estimated resident population living in private dwellings of Australia at 30 June 2011, based on the 2011 Census of Population and Housing [19]. For our analysis in Stata, the primary sampling units were set to ‘missing’ as the whole population sample was used, and variance estimation was performed using Taylor linearization.

**Figure 1:**
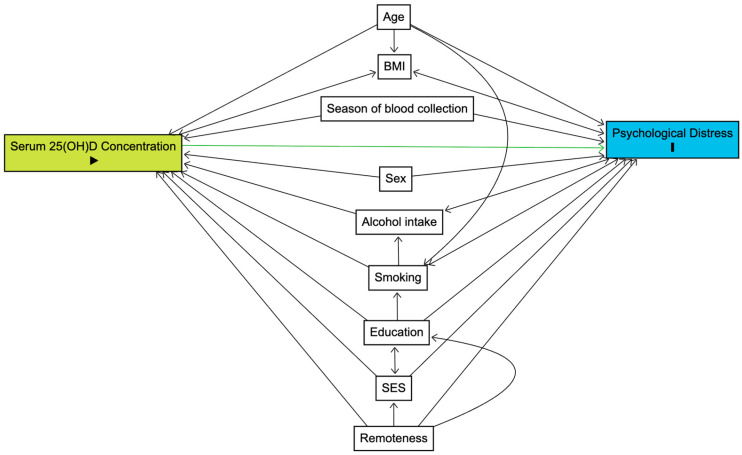
Factors associated with serum 25(OH)D concentration (play symbol: exposure) and psychological distress (I symbol: outcome). The green line indicates the potential associative path, solid black lines indicate a possible association, and arrows indicate the likely direction of the association. 25(OH)D, 25-hydroxyvitamin D; BMI, body mass index; SES, socioeconomic status.

We generated a directed acyclic graph using the ‘R’ package, ‘dagitty’ [29], to guide the selection of covariates (Figure 1), resulting in the following covariates: age, sex, education, remoteness, socioeconomic status, season of blood collection, alcohol intake, and smoking. Given the potential bidirectional relationships between body mass index and serum 25(OH)D concentration [30,31] and mental health [32,33,34,35], body mass index was not included as a covariate. The continuous covariates (age and alcohol intake) were tested visually for normality by plotting a frequency graph. Descriptive statistics were used to summarise participant characteristics: continuous variables were reported as mean (standard deviation [SD]) for parametric data and median (25th, 75th percentile) for non-parametric data; categorical variables were reported as frequency and percentages, *n* (%). 

We included participants with complete data for exposure, outcome, and covariates. Binary logistic regression was used to test associations between serum 25(OH)D concentration and K5 using two models: (i) unadjusted; and (ii) adjusted for age, sex, education, remoteness, socioeconomic status, season of blood collection, alcohol intake, and smoking. We assessed the multicollinearity of the exposure and all covariates using the variance inflation factor; significant multicollinearity was indicated if the variance inflation factor was > 10 [36]. The final model fit was assessed using the Hosmer–Lemeshow test. Given that serum 25(OH)D concentration differs by sex [37] and remoteness [6], and psychological distress differs by sex [38] and remoteness [38], we conducted exploratory analyses stratified by sex and by remoteness. Statistical significance was defined as *p* < 0.05.

## 3. Results

Of the 9317 participants of the 2012-2013 NATSIHS, 2060 participated in the biomedical component (i.e., provided blood samples for the measurement of serum 25(OH)D concentration). Complete data for exposure, outcome, and covariates were provided by a total of 1983 participants, of which 799 were males and 1184 were females; 915 lived in non-remote areas and 1068 in remote areas. Characteristics of the participants are presented in Table 1.

In the total population, there was no statistically significant association between serum 25(OH)D concentration and K5 in either the unadjusted or adjusted model (Table 2). There was good fit in the adjusted model (Hosmer–Lemeshow; χ^2^(8) = 3.98, *p* = 0.859).

In stratified analyses, we found a significant inverse association between serum 25(OH)D concentration and K5 among females in the adjusted model: each 10 nmol/L increase in serum 25(OH)D concentration was associated with 10% lower odds of high/very high psychological distress. There were no statistically significant associations between serum 25(OH)D concentration and K5 in males. When stratified by remoteness, there was a significant inverse association between serum 25(OH)D concentration and K5 among those living in remote areas: in the adjusted model, each 10 nmol/L increase in serum 25(OH)D concentration was associated with 11% lower odds of high/very high psychological distress. There were no statistically significant associations between serum 25(OH)D concentration and K5 among those living in non-remote areas.

## 4. Discussion

Using nationally representative data from the Australian Aboriginal and Torres Strait Islander population, we found no association between serum 25(OH)D concentration and psychological distress in the total population. However, when stratified by sex, higher serum 25(OH)D concentration was significantly associated with lower odds of high/very high levels of psychological distress among females, but not males. When stratified by remoteness, higher serum 25(OH)D concentration was significantly associated with lower odds of high/very high levels of psychological distress among those living remotely only. These findings should be interpreted with caution, given the cross-sectional study design and the use of the short K5 questionnaire as a non-specific measure of psychological distress, rather than a diagnosed mental health outcome. Nevertheless, as the first study examining associations between serum 25(OH)D concentration and mental health among Aboriginal and Torres Strait Islander peoples in Australia, the findings provide some foundational data on the relationship between vitamin D and mental health among this unique population group.

Studies of the general Australian population have shown mixed findings in relation to serum 25(OH)D concentration and mental health. Using data from the Western Australian Pregnancy Cohort (Raine) Study, our team previously reported an inverse association between serum 25(OH)D concentration and symptoms of depression, but not anxiety and stress, among young adult males but not females [39]. Findings from the Australian Safe-D study showed no association between serum 25(OH)D concentration and mental health (assessed by K10) in young women [40].

Internationally, population-based studies have also shown mixed findings. There was no association between circulating 25(OH)D concentration and mental health in population-based studies conducted in older adults in China [16], adults in the United States [15], and adults in Denmark [17]. In contrast, using data from a population-based study of adults in Finland, higher serum 25(OH)D concentration was associated with lower risks of depressive disorder and major depressive disorder, but not with depressive symptoms or anxiety disorder [13]. Further, lower serum 25(OH)D concentration was associated with higher depression severity in older adults in the Netherlands [12], and lower serum 25(OH)D concentration was associated with higher risk of depression in an adult Norwegian population, with a stronger association in women than men [14].

It is not clear why we found that higher serum 25(OH)D concentration was associated with lower odds of high/very high levels of psychological distress among those living in remote, but not non-remote, regions. There is a higher prevalence of serum 25(OH)D concentrations of < 50 nmol/L among Aboriginal and Torres Strait Islander peoples living in remote (39%) compared to non-remote areas (23%), and it has been postulated that changes to clothing and housing structure since colonisation may limit sun exposure in remote areas [6,22]. While vitamin D can be obtained from food and supplements, dietary vitamin D intake did not differ between remote and non-remote areas in our previous analysis of the 2012–2013 National Aboriginal and Torres Strait Islander Nutrition and Physical Activity Survey [41], and supplement use was low across the Aboriginal and Torres Strait Islander population [42]. In a previous analysis of the AATSIHS, the proportion of adults reporting high/very high levels of psychological distress was lower in those living in remote areas (24%) compared to non-remote areas (32%) [38]. Aboriginal and Torres Strait Islander peoples living in remote areas may have a strong supportive community network for individuals to share their hardships, which may have beneficial impacts on their mental health [43]. While there are differences in the exposure and outcome among those living remotely and non-remotely, our findings may be due to chance.

The strengths of our study include the use of nationally representative data for the Aboriginal and Torres Strait Islander population, and that serum 25(OH)D concentration was measured using an LC-MS/MS assay that was certified to international reference measurement procedures [19,20]. The AATSIHS included comprehensive data on socioeconomic and lifestyle characteristics, allowing us to adjust for potential confounders. However, the outcome measure of K5 is a non-specific measure of psychological distress and is not a diagnostic tool [26,44]. For Aboriginal and Torres Strait Islander peoples, mental health and wellbeing are a combination of various factors, such as their cultural identity, relationships with others, and community [3], which is not captured by the K5. Further, given the sensitive nature of the content of the K5, underreporting may have occurred if participants were interviewed in the presence of other household members. In addition, participants living remotely may have been reluctant to provide an accurate response to an individual outside their trusted community [19]. A further limitation was the age of the survey data (2012–2013); however, these are the most recent nationally representative data available for this population group. Due to the cross-sectional study design, causality cannot be inferred, and residual confounding by unmeasured characteristic cannot be ruled out.

## 5. Conclusions

In this exploratory study, we found no association between serum 25(OH)D concentration and psychological distress among the total population. Higher serum 25(OH)D concentration was associated with lower odds of high/very high psychological distress among females and among those living remotely; however, this finding should be interpreted with caution, as they are exploratory and may be due to chance. While our findings in relation to mental health are exploratory, given the high prevalence of low vitamin D status among this population, promoting adequate vitamin D status remains an important public health issue.

## Figures and Tables

**Table 1:** Characteristics of Aboriginal and Torres Strait Islander peoples aged ≥ 18 years included in the present study (*n* = 1983) ^1^.

Characteristics	Results
Age (years), median (25th, 75th percentile)	36 (26, 48)
Sex, *n* (%)	
Male	799 (48.6)
Female	1184 (51.4)
Serum 25(OH)D concentration (nmol/L), mean (SD)	64.0 (22.2)
Category of 25(OH)D, *n* (%) ^2^	
25(OH)D ≥ 50 nmol/L	1272 (72.2)
25(OH)D 30–49 nmol/L	575 (22.8)
25(OH)D < 30 nmol/L	136 (5.0)
K5 score, median (25th, 75th percentile)	9 (6, 13)
Category of K5, *n* (%) ^2^	
Low/moderate psychological distress (5–11)	1386 (68.5)
High/very high psychological distress (12–25)	597 (31.5)
Smoking status, *n* (%)	
Ex/non-smoker	1117 (64.2)
Current smoker	866 (35.8)
Alcohol intake, median (25th, 75th percentile) ^3^	0.5 (0, 5.8)
Education, *n* (%)	
No/primary/high school	1164 (51.3)
Diploma/certificate	681 (42.0)
University	138 (6.7)
Socioeconomic status, *n* (%)	
Quintile 1	1187 (48.5)
Quintile 2	348 (23.0)
Quintile 3	200 (12.2)
Quintile 4	181 (12.6)
Quintile 5	67 (3.7)
Remoteness, *n* (%)	
Non-remote	915 (77.8)
Remote	1068 (22.2)
Season of blood collection, *n* (%)	
Summer	392 (27.8)
Autumn	110 (9.4)
Winter	529 (24.5)
Spring	952 (38.3)

25(OH)D, 25-hydroxyvitamin D; K5, Kessler Psychological Distress Scale 5; SD, standard deviation. ^1^ Weighted to the Aboriginal and Torres Strait Islander estimated resident population living in private dwellings of Australia at 30 June 2011, based on the 2011 Census of Population and Housing. ^2^ Categories as reported by the Australian Bureau of Statistics. ^3^ Average daily intake based on standard drinks consumed over the last three drinking days.

**Table 2:** Associations between serum 25(OH)D concentration (per 10 nmol/L) and K5 among Aboriginal and Torres Strait Islander peoples aged ≥ 18 years ^1^.

	Unadjusted				Adjusted ^2^			
	β	OR	95% CI	*p*-Value	Adj. β	Adj. OR	95% CI	*p*-Value
Total population (*n* = 1983)	−0.07	0.93	−0.16, 0.02	0.119	−0.08	0.92	−0.17, 0.01	0.088
Stratified by sex:								
Male (*n* = 799)	−0.06	0.94	−0.21, 0.09	0.457	−0.02	0.98	−0.17, 0.13	0.805
Female (*n* = 1184)	−0.08	0.92	−0.18, 0.02	0.136	−0.11	0.90	−0.23, −0.003	0.045
Stratified by remoteness:								
Non-remote (*n* = 915)	−0.07	0.93	−0.17, 0.03	0.160	−0.08	0.92	−0.20, 0.03	0.150
Remote (*n* = 1068)	−0.11	0.90	−0.21, −0.01	0.033	−0.12	0.89	−0.22, −0.01	0.029

25(OH)D, 25-hydroxyvitamin D; β, beta coefficient; Adj. β, adjusted beta coefficient; Adj. OR, adjusted odds ratio; CI, confidence interval; K5, Kessler Psychological Distress Scale 5; OR, odds ratio. ^1^ Weighted to the Aboriginal and Torres Strait Islander estimated resident population living in private dwellings of Australia at 30 June 2011, based on the 2011 Census of Population and Housing. ^2^ Adjusted for age, sex, education, remoteness, socioeconomic status, season of blood collection, alcohol intake, and smoking.

## Data Availability

Data are available in ABS Data Laboratory environment, https://www.abs.gov.au/ausstats/abs@.nsf/PrimaryMainFeatures/4715.0.30.001?OpenDocument (accessed on 16 October 2024).

## Abbreviations

The following abbreviations are used in this manuscript:

25(OH)D	25-hydroxyvitamin D
AATSIHS	Australian Aboriginal and Torres Strait Islander Health Survey
ABS	Australian Bureau of Statistics
K5	Kessler Psychological Distress Scale 5
LC-MS/MS	Liquid Chromatography with Tandem Mass Spectrometry
NATSIHS	National Aboriginal and Torres Strait Islander Health Survey
